# Shen-Shuai-Ning granule decreased serum concentrations of indoxyl sulphate in uremic patients undergoing peritoneal dialysis

**DOI:** 10.1042/BSR20171694

**Published:** 2018-09-14

**Authors:** Xujiao Chen, Shouhong Gao, Mengna Ruan, Sixiu Chen, Jing Xu, Xiaohong Xing, Xiaohong Pan, Changlin Mei, Zhiguo Mao

**Affiliations:** 1 Kidney Institute of CPLA, Division of Nephrology, Changzheng Hospital, Second Military Medical University, Shanghai, China; 2 Department of Physiology and Biomedical Engineering, Mayo Clinic, Rochester, MN, U.S.A.; 3 Department of Pharmacy, Changzheng Hospital, Second Military Medical University, Shanghai, China

**Keywords:** Indoxyl sulfate, P-cresyl sulfate, Peritoneal dialysis, Shen-Shuai-Ning

## Abstract

Clearance of protein-bound uremic toxins (PBUTs) by dialysis is a challenge in the treatment of uremic patients. Shen-Shuai-Ning (SSN), a traditional Chinese medicine formulation, has been used commonly in China to retard kidney disease progression and decrease uremic toxins in chronic kidney disease (CKD) patients, but the effects of SSN on serum PBUTs in dialysis patients were not investigated. We conducted a randomized controlled trial in patients on peritoneal dialysis (PD) at dialysis center of Changzheng Hospital to evaluate the effects of SSN on serum PBUTs. Participants with SSN intervention took 5 g SSN granule three times daily for 12 weeks, while the baseline medications and dialysis prescriptions remained during the study in all patients. The serum concentrations of indoxyl sulphate (IS) and *p*-cresol sulphate (PCS) were determined by HPLC/MS/MS and biochemical parameters were assessed during the study. Sixty PD patients were enrolled and randomly allocated into SSN group and control group. Total IS level was significantly lower in SSN group than in control group at week 4, 8, and 12 (27.28 ± 18.19, 29.73 ± 19.10, and 29.41 ± 17.61 mg/l compared with 39.25 ± 20.23, 44.86 ± 23.91, and 45.34 ± 33.52 mg/l, respectively). However, there were no statistical difference of total PCS, free forms of IS and PCS concentrations between SSN group and control group during 12 weeks follow-up. Administration of SSN granule orally decreased serum total IS level effectively in uremic patients on PD with good tolerance. Benefits of PD patients’ outcomes from IS reduction by SSN awaits further large size and long duration clinical trials to verify.

## Introduction

Chronic kidney disease (CKD) is associated with dramatically increased risk of cardiovascular disease due to complex pathophysiologic responses to declining kidney function [1–3]. As CKD evolves, numerous metabolites accumulate gradually due to altered metabolism and/or reduced renal clearance, and become a progressively more important contributor to cardiovascular dysfunction [4–6]. Amongst the uremic solutes, the pathophysiologic importance of protein-bound uremic toxins (PBUTs) has drawn increasingly extensive attentions. Unlike other water-soluble uremic toxins, PBUTs cannot be effectively removed by available dialysis options in patients with end-stage renal disease (ESRD).

In the past decade, a growing number of publications suggested that indoxyl sulphate (IS) and *p*-cresol sulphate (PCS), two types of most investigated PBUTs, had direct deleterious effects on a variety of organs. They could inhibit blood vessel endothelial proliferation and migration [7], increase ROS, and decrease NO production [8] leading to endothelial dysfunction. Previous studies documented the negative impact of IS and PCS on vital processes and an association of their concentration with the poor long-term outcome of dialysis or pre-dialysis CKD patients [5,9,10].

Shen-Shuai-Ning (SSN), a traditional Chinese medicine formulation, has been used for decreasing serum creatine in pre-dialysis patients for many years [11]. SSN is composed of *Rheum officinale* (Da Huang), *Radix pseudostellariae* (Tai Zi Shen), *Coptis chinensis* (Huang Lian), *Carthamus tinctorius* (Hong Hua), the rhizome of *Salvia miltiorrhiza* (Danshen), and *Bidentate achyranthes* (Niu Xi)*.* By fastening colonic transit, decreasing the absorption of toxins, SSN could reduce the levels of serum creatine and delay the initiation of dialysis in patients with CKD [11,12]. However, the effect of SSN on reducing PBUTs in peritoneal dialysis (PD) patients has rarely been investigated.

## Materials and methods

### Subjects

Stable ESRD patients receiving PD were recruited from 1 February 2015 to 28 February 2016 in dialysis center of Changzheng Hospital, Shanghai, China. Patients were excluded when they were: older than 75 or younger than 18 years; dialysis duration was less than 3 months; with PD-related peritonitis or other infectious diseases in the past 1 month; at the active stage of autoimmune diseases; suffering malignancies; cardiovascular events in the past 6 months; allergic to SSN; and taking intestinal adsorbent drugs or antibiotics in the past 1 month. All fertile female patients were required to take contraception methods during study. This clinical trial was approved by the Ethics Committee of Changzheng Hospital and informed consent was obtained from all patients. The clinical trials registry number was ChiCTR-IOR-14005541.

Cardiovascular events were defined as: acute myocardial infarction, percutaneous transluminal coronary angioplasty, cardiovascular accident, or transient ischemic attack.

Randomization sequences were computer generated and eligible patients were randomly assigned to SSN group or control group by using a 1:1 ratio.

SSN granule was administrated orally in SSN group for 12 weeks, while the baseline medications and dialysis prescriptions maintained the same in the SSN group and control group. SSN granule was given with a dosage of 5 g three times daily after meals, 1 h apart from other drugs.

### Study drug

The SSN granule was manufactured by Deyuantang Industry Co. Ltd, Shanxi, China. To verify the stability of SSN granule’s composition, ten different batches of SSN granules were tested using HPLC method. A 100 mg SSN granule was added into 2 ml methanol ultrasonic extraction and incubated for 30 min, then filtered through a 0.45-μm nylon membrane filter for HPLC analysis. Chromatographic analysis was performed on an Agilent 1260 series HPLC instrument (Agilent, Inc., MA, U.S.A.) coupled to a binary pump (G1311C), an autosampler (G1329B), a thermostatted column compartment (G1316A), and a UV detector (G4212B-DAD). The sample was separated on a SunFire™ C18 (250 mm × 4.6 mm, 5 μm) with the column temperature maintained at 25°C. The analytical separation was run using a gradient elution composed of solvent A (acetonitrile) and solvent B (0.1% formic acid). A linear gradient elusion was used as follows: 0–1 min, 5–23% A; 1–18 min, 23–25% A; 18–19 min, 25–30% A; 19–31 min, 30–75% A; 31–60 min, 75–85% A; 60–90 min, 85–100% A. The flow rate was 1.0 ml/min and the UV spectra were set at 254 nm. The injection volume was 5 μl.

### Parameters

The serum concentrations of total and free IS and PCS were determined by HPLC/MS/MS method, as we prescribed previously [13], at baseline, weeks 4, 8, and 12. Briefly, 200 μl acetonitrile containing DHCT (2 μg/ml) was added to 100 μl serum sample, centrifuged at 13400 rpm for 5 min, and the supernatant was equally mixed (1:1, v/v) with 10 mM ammonium acetate buffer for analysis. Free IS and PCS was measured with the same method, except that serum samples were ultrafiltered through a centrifugal filter (MWCO 30000Da, Millipore) before detection. HPLC/MS/MS analysis was carried out on an Agilent 1200 series HPLC interfaced to an Agilent 6410A triple-quadrupole mass spectrometer equipped with an ESI source (Agilent Inc, MA, U.S.A.). The separation was performed on an Agilent Zorbax SB-C18 column (3.5 μm, 2.1 mm × 100 mm) with the column temperature maintained at 30°C. The mobile phase consisted of a mixture of acetonitrile and 10 mM ammonium acetate buffer (10:90, v/v) using an isocratic elution at a flow rate of 0.3 ml/min. A 5-μl aliquot of sample solution was injected and analyzing time of each injection was 5 min. Quantitation was performed using electrospray in the negative mode with the spray voltage was set at 4000 V. *p*-cresyl sulphate ammonium salt with a purity >98.5% (Alsachim, Strasbourg, France), IS potassium salt, and hydrochlorothiazide with a purity >98% (Melone Pharmaceutical Co., Ltd, Dalian, China) were used as internal. The linearity ranged from 500 to 10000 ng/ml for IS (*r* > 0.99) and 50 to 10000 ng/ml for PCS (*r* > 0.99). The limit of detection was 500 ng/ml for IS and 50 ng/ml for PCS. Relative S.D. of intra- and interday precision were within ±15%.

Demographic data including underlying renal disease, age, gender, duration of dialysis therapy, and co-morbidity were reviewed and recorded. Blood pressure was measured on their monthly clinics. Biochemical parameters of all patients were measured at baseline and the end of study. The adequacy of PD, including total Ccr and Kt/V, and residual renal function (RRF) were obtained. Patients with daily urine output less than 100 ml were regarded as non-RRF.

### Statistical analysis

All values are presented as mean ± S.D. Statistical analysis was performed using SPSS 20.0 for Windows (SPSS, Inc., Chicago, IL, U.S.A.). For statistical analysis, Student’s *t* test and χ^2^ test were used to compare categorical and continuous data between the SSN and control groups at baseline, weeks 4, 8, and 12.

## Results

Sixty PD patients from PD Center of Changzheng Hospital were recruited between 1 February 2015 and 28 February 2016, and 30 of them were randomized to SSN group and 30 to control group. Mean age was 46.23 years and 73.3% were males in SSN group, while 45.57 years and 60.0% in control group. The causes of ESRD, duration of PD, body mass index (BMI), and blood pressure were comparable between two groups (Table 1).

**Table 1:** Baseline characteristics of the patients in SSN and control groups

	SSN group (*n*=30)	Control group (*n*=30)	*P*
Age (years)	46.23 ± 11.21	45.57 ± 12.41	0.83
Males	22 (73.3%)	18 (60.0%)	0.41
Duration of PD (months)	18.77 ± 13.19	17.57 ± 17.92	0.77
MAP (mmHg)	99.18 ± 14.17	103.72 ± 12.43	0.20
BMI (kg/m^2^)	22.70 ± 3.11	22.37 ± 3.24	0.69
Cause of ESRD			
CGN	18 (60.0%)	16 (53.4%)	0.85
Diabetes	2 (10.0%)	4 (13.3%)	
Hypertensive nephropathy	4 (10.0%)	4 (13.3%)	
Others	6 (20%)	6 (20.0%)	

Abbreviations: CGN, chronic glomerulonephritis; MAP, mean arterial pressure.

### Test of SSN granule samples

**Figure 1:**
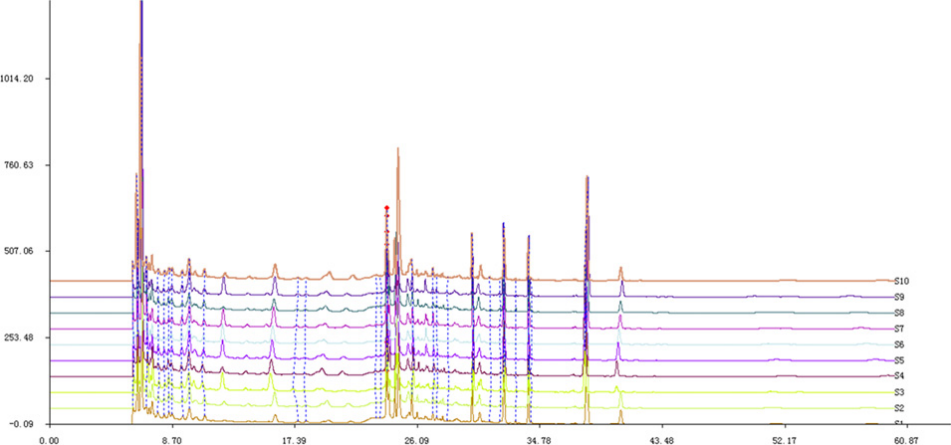
Ten different batches of SSN granules were tested using HPLC/MS/MS method S1–10, ten stands for ten batches of SSN (numbers 51103111, 51103009, 51103010, 51103011, 51103105, 51103018, 41103033, 51103110, 61103102, and 61103101).

Ten different batches (batch numbers 51103111, 51103009, 51103010, 51103011, 51103105, 51103018, 41103033, 51103110, 61103102, and 61103101) of SSN granules were tested by HPLC method and the results showed the composition stability of SSN granules (Figure 1). All of the chromatograms’ results were imported into the ‘Similarity Evaluation System for Chromatographic Fingerprints of Traditional Chinese Medicine’ (Chinese Pharmacopoeia Commission, version 2009). The similarity scores of common peaks from these samples ranged from 0.867 to 1.000 (Supplementary Table S1), indicating that samples from different batches were highly correlated.

### Comparison of serum biochemical parameters between two groups

There were no statistical differences in biochemical parameters, including lips, liver function, hemoglobin (Hb), calcium, phosphorus, and intact parathyroid hormone (iPTH), between two groups either at baseline or at the end of the study (Supplementary Table S2).

**Table 2:** Serum concentrations of IS and PCS at baseline, weeks 4, 8, and 12 in SSN and control groups

		SSN group	Control group	*P*
**Baseline**		*n*=30	*n*=30	
	Total IS (mg/l)	32.33 ± 20.18	29.40 ± 16.85	0.54
	Free IS (mg/l)	2.94 ± 2.88	1.99 ± 1.90	0.14
	Total PCS (mg/l)	23.90 ± 19.87	17.90 ± 15.03	0.19
	Free PCS (mg/l)	2.03 ± 1.92	1.41 ± 1.14	0.14
**4 weeks**		*n*=28	*n*=30	*P*
	Total IS (mg/l)	27.28 ± 18.19	39.25 ± 20.23	0.02
	Free IS (mg/l)	2.23 ± 2.42	2.76 ± 2.70	0.44
	Total PCS (mg/l)	18.85 ± 13.79	26.87 ± 22.99	0.12
	Free PCS (mg/l)	1.72 ± 1.68	2.08 ± 2.52	0.53
**8 weeks**		*n*=26	*n*=29	*P*
	Total IS (mg/l)	29.73 ± 19.10	43.86 ± 23.91	0.02
	Free IS (mg/l)	2.54 ± 3.39	3.27 ± 2.33	0.36
	Total PCS (mg/l)	13.47 ± 8.50	17.60 ± 12.36	0.16
	Free PCS (mg/l)	1.03 ± 1.16	1.49 ± 1.46	0.21
**12 weeks**		*n*=25	*n*=26	*P*
	Total IS (mg/l)	29.41 ± 17.61	45.34 ± 33.52	0.04
	Free IS (mg/l)	2.40 ± 2.26	3.49 ± 2.53	0.11
	Total PCS (mg/l)	19.12 ± 16.31	20.73 ± 12.94	0.70
	Free PCS (mg/l)	1.45 ± 1.35	1.49 ± 1.37	0.70

### Profile of serum IS and PCS

At baseline, total IS concentrations were 32.33 ± 20.18 and 29.40 ± 16.85 mg/l (*P*=0.54) and total PCS were 23.90 ± 19.87 and 17.90 ± 15.03 mg/l (*P*=0.19) in SSN group and control group, respectively. The two groups were not different in the levels of total and free forms of serum IS and PCS.

With time, the serum total IS concentration in SSN group decreased to 27.28 ± 18.19 mg/l at week 4 and kept stable until week 12, meanwhile, total IS levels in control group did not decrease and the difference of total IS levels between two groups was statistically significant from week 4 to the end of the intervention. However, the serum total PCS concentration in SSN group did not show statistical difference from that in control group through the study.

**Figure 2:**
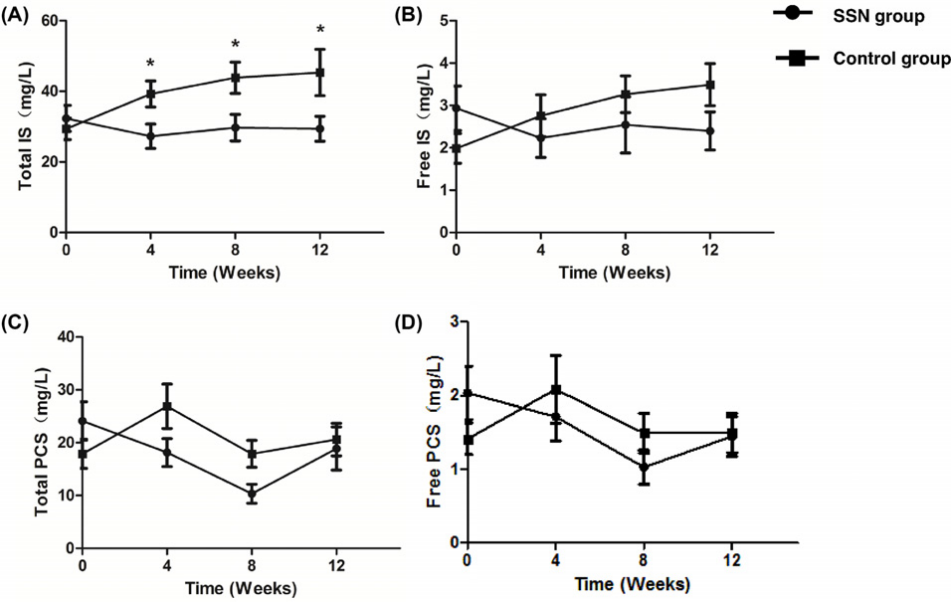
Serum concentration of PBUTs (mean ± S.E.M.) in two groups at baseline and at weeks 4, 8, and 12 Serum total IS level was lower in SSN group than in control at weeks 4, 8, and 12 (**A**). The serum-free form of IS (**B**) and PCS (**D**) did not showed obvious differences during the study between two groups. (**C**) The serum total PCS in SSN group showed marginal trends of lower levels in SSN group than control group at weeks 4 and 8 although the differences were not statistically significant (D). *, *P*<0.05.

The serum-free forms of both IS and PCS did not show difference between two groups during the intervention (Table 2 and Figure 2).

### Changes in RRF and other biochemical data

There was no statistical difference in RRF, daily urine output, and solute dialysis adequacy between SSN group and control group at baseline and at week 12 of the study (Table 3).

**Table 3:** Dialysis adequacy and RRF in SSN and control groups at baseline and the end of study

	Baseline			Week 12		
	SSN group (*n*=30)	Control group (*n*=30)	*P*	SSN group (*n*=25)	Control group (*n*=26)	*P*
**RRF (ml/min)**	2.76 ± 2.27	2.46 ± 3.03	0.66	2.23 ± 1.91	1.98 ± 3.33	0.74
**Kt/v (l/week)**	1.72 ± 0.36	1.78 ± 0.39	0.51	1.69 ± 0.45	1.72 ± 0.38	0.77
**Ccr (l/week)**	64.42 ± 18.56	62.42 ± 27.46	0.75	60.03 ± 14.38	59.86 ± 27.33	0.98
**24-h urine volume (ml)**	866.55 ± 546.24	806.86 ± 747.40	0.73	696.40 ± 415.07	606.54.50 ± 567.94	0.52

## Discussion

Several lines of clinical evidence suggested that serum IS and PCS levels were associated with poor clinical outcomes in ESRD patients [14–17]. In hemodialysis populations, serum PCS and IS levels were shown to be associated with heart failure and other cardiovascular events [14,16], and in PD patients, serum total PCS was associated with cardiovascular and mortality events, and IS and PCS were valuable in PD failure prediction [17]. In pre-dialysis CKD patients, serum IS level was reported to predict cardiovascular disease and renal function decline [15].

IS and PCS are known to be gut-derived uremic toxins and normally cleared by the kidneys. In gut, bacterial metabolism of tryptophan results in the formation of indole, which is oxidized to indoxyl after absorption and finally sulphated to IS. Similarly, bacterial fermentation of tyrosine leads to *p*-cresol, after intestinal absorption is sulphated, resulting in the formation of PCS. The interaction between intestinal absorption of bacterial metabolites, human metabolism, and the urinary excretion has been named the gut–kidney axis [18,19]. Intestinal adsorption and dialysis clearance are two determinants for the accumulation of some uremic toxins, like IS and PCS, in ESRD patients.

Due to the high protein-binding rate of IS and PCS, the clearance capacity by available dialysis options are limited [20,21]. Studies have shown the importance of gut microbiota dysbiosis in ESRD patients, indole- and *p*-cresol-forming bacterial families were found to be more abundant in ESRD patients, which could lead to increased IS and PCS production [22–25]. Meanwhile, inhibition of the production of the uremic toxins precursors in gut may be an effective way to reduce the accumulation of *p*-creyl sulphate and IS.

The efficacy of traditional Chinese medicines or their active ingredients was proved in many diseases such as malaria in recent years [26]. SSN is traditionally orally used for the treatment of CKD for pre-dialysis patients in China, for the purpose of retarding the progression of kidney disease and lowering the serum toxins such as creatinine. Amongst the formulation of SSN, *R. officinale*, also called Da Huang in Chinese, can increase bowel movements, promote excretion of toxic metabolites, such as blood urea nitrate and creatinine from gut [27]. Some authors proved *R. officinale* might reduce intestine-derived uremic toxins produced by gut bacteria through regulating the intestinal environment [28,29]. *S. miltiorrhiza*, known as Danshen, showed significant renal protective effects in iron-overloaded animal models by decreasing iron deposition and inhibiting lipid peroxidation and apoptosis in the kidney [30].

As the quality of Chinese herbal medicines can be influenced by the herb’s origin, the climate and season when the herb was grown and harvested, the instability of herbal ingredients is often a concern. In the present study, ten randomized batches of SSN granules during the study period were tested by HPLC/MS/MS methods and the repeatable results showed that the compositions of this drug were stable under standardized manufacturing quality control.

Our data suggested that SSN decreased the total serum IS concentrations in PD patients. To our best knowledge, this pilot study is the first one to explore the effects of traditional Chinese medicine on the PBUTs in PD patients. The present study showed that treatment with the oral SSN granule reduced the serum concentration of total IS significantly after 4 weeks of intervention, and had a trend to decrease the serum concentration of total PCS at weeks 4 and 8, suggesting the efficacy of SSN in removing PBUTs in patients undergoing PD. We speculate that SSN reduces the production and absorption of uremic toxins or their precursors in the gastrointestinal tract by improving intestinal barrier function, accelerating intestinal dynamics, increasing the frequency of defecation, and perhaps regulating intestinal flora.

Studies showed that serum IS level was a valuable marker in predicting kidney function decline in patients with advanced CKD [15,31,32], implying the lower levels of IS might be associated with slower loss of RRF. However, in this study we did not find the association between lower serum IS levels and better RRF reservation in the 12-week follow-up. The short observation duration and small sample size might be the explanation.

The major concern on SSN granule in PD patients was the gastrointestinal reactions, including nausea and diarrhea. During the study, four patients in SSN group dropped out due to the intolerance to the proposed dose of SSN granule (5 g, three times daily). After dropout, two patients resumed SSN with a lower dose (5 g, once or twice daily) and showed good tolerance. However, the overall number of adverse events in two groups were comparable, implying the possible benefits from serum PBUT reduction by SSN might indirectly decrease the risks of other systems.

There are some limitations of our study. First, the sample size of the study was small and all subjects were enrolled in a single PD center. Second, due to the unique smell and flavor of SSN granule, blind design of the study was not feasible, which might weaken the reliability of the results. Third, the study duration was 12 weeks and that was probably why we did not find obvious clinical benefits of serum IS reduction in PD patients, such as RRF preservation. Whether lowering the serum levels of IS by SSN granule can improve the clinical outcomes of PD patients could not be answered by the present study.

In conclusion, the present study explored the effects of SSN, a traditional Chinese medicine formulation, on PBUTs in ESRD patients undergoing PD. The present data showed that SSN reduced serum total IS levels with good tolerance. As IS was associated with cardiovascular events, RRF loss, and mortality in CKD patients, whether administration of SSN will improve the clinical outcomes of PD patients awaits larger sample, long follow-up, and multicenter prospective studies to further verify.

## Clinical perspectives

IS and PCS, two types of most investigated PBUTs, had a direct deleterious effect on a variety of organs. Previous studies documented the negative impact of IS and PCS on vital processes and an association of their concentrations with the poor long-term outcome of dialysis or pre-dialysis CKD patients. Unlike other water-soluble uremic toxins, PBUTs cannot be effectively removed by available dialysis options in patients with ESRD. SSN, a traditional Chinese medicine formulation, has been used for decreasing serum creatine in pre-dialysis patients for many years. Through increasing the cacation, decreasing the absorption of toxins, SSN could reduce the levels of serum creatine and delay the initiation of dialysis in patients with CKD. However, the effect of SSN on reducing PBUTs in PD patients has rarely been investigated. Our data suggested that (1) SSN decreased the total serum IS concentrations in PD patients and (2) reducing the serum IS level may probably improve the clinical outcomes in these patients.

## Supporting information

**Supplement table 1:** SimilarityEvaluation of tenten batches batches SSN

## Abbreviations

Ccr: creatinine clearance rate
CKD: chronic kidney disease
DHCT: dihydrochlorothiazide
ESRD: end-stage renal disease
IS: indoxyl sulphate
MWCO: molecular weight cut off
PBUT: protein-bound uremic toxin
PCS: *p*-cresol sulphate
ROS: reactive oxygen species
RRF: residual renal function
SSN: Shen-Shuai-Ning
