# Supplementary material for: Shen-Shuai-Ning granule decreased serum concentrations of indoxyl sulphate in uremic patients undergoing peritoneal dialysis
Source: Biosci Rep. 2018 Sep 14;38(5):BSR20171694. doi: 10.1042/BSR20171694 (PMC6137246; doi:10.1042/BSR20171694)
Supplement: Supplementary file 1 [file bsr20171694_Supp1.pdf]

Supplement table 1. Similarity Evaluation of ten batches SSN

Batch No.	S1	S2	S3	S4	S5	S6	S7	S8	S9	S10	Comparison
**S1**	1	0.98	0.91	0.941	0.934	0.948	0.916	0.941	0.95	0.874	0.965
**S2**	0.98	1	0.934	0.934	0.932	0.945	0.951	0.946	0.978	0.867	0.973
**S3**	0.91	0.934	1	0.952	0.947	0.937	0.972	0.94	0.945	0.914	0.973
**S4**	0.941	0.934	0.952	1	0.99	0.984	0.951	0.971	0.923	0.94	0.986
**S5**	0.934	0.932	0.947	0.99	1	0.978	0.938	0.976	0.91	0.91	0.979
**S6**	0.948	0.945	0.937	0.984	0.978	1	0.954	0.987	0.931	0.923	0.986
**S7**	0.916	0.951	0.972	0.951	0.938	0.954	1	0.949	0.966	0.913	0.979
**S8**	0.941	0.946	0.940	0.971	0.976	0.987	0.949	1	0.928	0.89	0.98
**S9**	0.95	0.978	0.945	0.923	0.91	0.931	0.966	0.928	1	0.892	0.969
**S10**	0.874	0.867	0.914	0.94	0.91	0.923	0.913	0.89	0.892	1	0.937
**Comparison**	0.965	0.973	0.973	0.986	0.979	0.986	0.979	0.98	0.969	0.937	1

Supplement table 2. Biochemical data in SSN and control groups at baseline and the end of study.

	Baseline			Week 12		
	SSN group (n=30)	Control group (n=30)	*P*	SSN group (n=25)	Control group (n=26)	*P*
**Creatinine (μmoI/L)**	1024.63±313.30	1043.63±306.6	0.813	1026.20±368.66	1130.50±277.88	0.258
**BUN (mmol/L)**	22.23±7.94	20.68±5.68	0.395	22.60±5.76	21.01±5.34	0.311
**WBC (10^9 / L)**	6.24±1.61	6.84±2.43	0.280	6.49±1.75	7.23±3.64	0.363
**Hb (g/L)**	102.13±19.16	100.64±24.95	0.803	103.32±17.27	102.50±16.52	0.863
**Plt (10^9 /L)**	207.28±73.97	174.37±50.65	0.060	203.08±65.26	206.89±82.66	0.856
**CRP (mg/L)**	6.76±11.67	7.54±14.34	0.884	9.92±12.83	7.88±9.61	0.523
**Albumin (g/L)**	35.32±5.62	36.08±5.71	0.626	35.68±5.32	36.70±5.51	0.501
**Calcium (mmol/L)**	2.28±0.24	2.26±0.33	0.827	2.28±0.21	2.34±0.21	0.348
**Phosphate (mmol/L)**	1.77±0.63	1.71±0.59	0.698	1.63±0.53	1.82±0.20	0.104
**iPTH (pg/ml)**	417.28±461.29	308.64±299.68	0.315	300.33±281.21	255.20±212.91	0.520
**TG (mmol/L)**	1.67±1.26	1.46±0.71	0.460	1.90±1.27	1.81±1.05	0.783
**CHOL (mmol/L)**	4.30±0.98	4.27±0.63	0.920	4.30±1.28	4.44±0.92	0.655
**LDL (mmol/L)**	2.76±0.77	2.65±0.69	0.599	2.34±0.91	2.28±0.60	0.780
**HDL (mmol/L)**	1.02±0.39	0.98±0.29	0.751	1.32±0.68	1.12±0.35	0.202
**AST (U/L)**	17.22±10.71	15.48±5.64	0.459	15.84±6.09	18.85±15.59	0.373
**ALT (U/L)**	18.56±14.02	15.74±6.38	0.347	15.24±7.76	21.38±29.32	0.311
**TBiL (μmoI/L)**	9.52±7.23	7.70±2.07	0.220	8.04±6.59	7.34±2.33	0.6166

Clinical Events and Drop-out

In SSN group, during the 12-weekstudy period, there were 2 patients withdrawn for nausea in 4 weeks; 1 patient withdrawn for nausea and 1 for diarrhea in 8 weeks; 1 patient turned to HD because of PD ultrafiltration failure in 12 weeks. In control group, 1 patient withdrawn for severe heart failure in 8 weeks; 2 patients withdrawn for PD-related peritonitis, 1 for severe heart failure in 12 weeks(Supplement table 3).

Supplement table 3. Severe adverse events of two groups in week 4, week 8 and week12.

	**Adverse events**	**SSN group**	**Control group**
Week 4	Nausea	2	0
Week 8	Nausea	1	0
	diarrhea	1	0
	Severe heart failure	0	1
Weeks 12	PD related peritonitis	0	2
	Turn to HD	1	0
	Severe heart failure	0	1
